# Identification and characterization of a calcium-binding peptide from salmon bone for the targeted inhibition of α-amylase in digestion

**DOI:** 10.1016/j.fochx.2024.101352

**Published:** 2024-04-03

**Authors:** Zhe Xu, Shiying Han, Na Cui, Hanxiong Liu, Xu Yan, Hongrui Chen, Jianping Wu, Zhijian Tan, Ming Du, Tingting Li

**Affiliations:** a College of Life Sciences, Key Laboratory of Biotechnology and Bioresources Utilization, Dalian Minzu University, Ministry of Education, Dalian 116600, China; b Department of Food and Chemical Engineering, Liuzhou Institute of Technology, Liuzhou, Guangxi 545616, China; c Institute of Bast Fiber Crops & Center of Southern Economic Crops, Chinese Academy of Agricultural Sciences, Changsha 410205, China; d Department of Agricultural, Food and Nutritional Science, University of Alberta, Edmonton, Alberta T6G2P5, Canada; e School of Food Science and Technology, National Engineering Research Center of Seafood, Collaborative Innovation Center of Seafood Deep Processing, Dalian Polytechnic University, Dalian 116034, China; f School of Food and Bioengineering, Food Microbiology Key Laboratory of Sichuan Province, Chongqing Key Laboratory of Speciality Food Co-Built by Sichuan and Chongqing, Xihua University, Chengdu, Sichuan 611130, China

**Keywords:** Targeted inhibition, α-amylase, Calcium-binding, Salmon bone, Peptide

## Abstract

α-Amylase, essential for carbohydrate digestion, relies on calcium (Ca) for its structural integrity and enzymatic activity. This study explored the inhibitory effect of salmon bone peptides on α-amylase activity through their interaction with the enzyme's Ca-binding sites. Among the various salmon bone hydrolysates, salmon bone trypsin hydrolysate (SBTH) exhibited the highest α-amylase inhibition. The peptide IEELEEELEAER (PIE), with a sequence of Ile-Glu-Glu-Leu-Glu-Glu-Glu-Glu-Leu-Glu-Ala-Glu-Arg from SBTH, was found to specifically target the Ca-binding sites in α-amylase, interacting with key residues such as Asp206, Trp203, His201, etc. Additionally, cellular experiments using 3 T3-L1 preadipocytes indicated PIE's capability to suppress adipocyte differentiation, and decreases in intracellular triglycerides, total cholesterol, and lipid accumulation. In vivo studies also showed a significant reduction in weight gain in the group treated with PIE(6.61%)compared with the control group (33.65%). These findings suggest PIE is an effective α-amylase inhibitor, showing promise for obesity treatment.

## Highlights

• Peptide IEELEELEAER(PIE) should target the inhibition of α-amylase activity.
• The site of calcium binding action of PIE in α-amylase by Discovery Studio.
• PIE should inhibit the differentiation of 3 T3-L1 and control body weight of mice.

## Graphical abstract

A calcium-binding peptide from salmon targeted inhibition of amylase in digestion.

**Figure:**
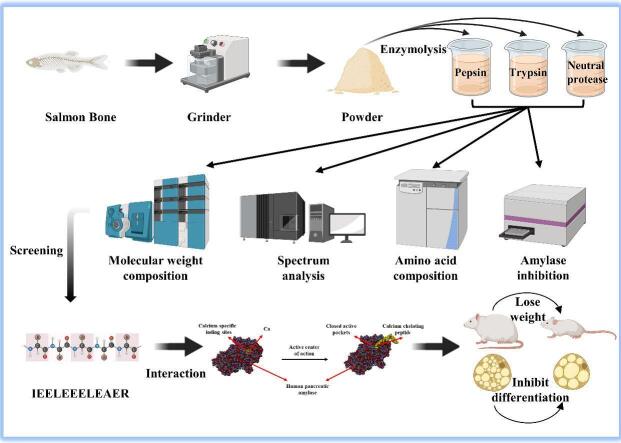

## 1 Introduction

The escalating incidence of obesity and diabetes has been linked to excessive carbohydrate consumption (Zhou et al., 2021). Carbohydrate digestion initiates in the mouth with the action of salivary amylase, which breaks down starch into simpler sugars. The process continues in the small intestine, where pancreatic α-amylase converts carbohydrates into dextrins and oligosaccharides. These are subsequently hydrolyzed into glucose, absorbed by the small intestine via sodium-dependent glucose transporter proteins and glucose transporters, and then distributed through blood circulation for energy metabolism (Akter et al., 2022). α-amylase inhibition impedes carbohydrate hydrolysis, thereby reducing sugar absorption, which in turn alleviates postprandial glycemic spike. This inhibition also increases the accumulation of undigested carbohydrates in the ileum and delays gastric emptying; thus, it enhances satiety and further reduces food intake (Saad et al., 2022). Hence, it can be used as an effective treatment for preventing and treating obesity and diabetes.

α-Amylase inhibitors are substances that inhibit the activity of the enzyme α-amylase, including compounds such as tea polyphenols, hydroxycitric acid, miglitol, etc. These inhibitors primarily delay glucose production during digestion through two mechanisms: (1) denaturation and inactivation of α-amylase by altering the structure of enzyme (Wee, Loud, Tan, & Forde, 2019), and (2) formation of an enzyme–inhibitor complex that prevents enzyme-substrate binding (Sun, Wang, & Miao, 2020). Based on enzyme reaction kinetics, the type of inhibition of the enzyme by the inhibitor can be classified into reversible and irreversible inhibition. In reversible inhibition, the enzyme and inhibitor bind through noncovalent bonds, resulting in a reduction or loss of enzymatic activity, but the enzyme can be revived once the inhibitor is removed (Qiu et al., 2020). In irreversible inhibition, the enzyme and inhibitor bind through covalent bonds, and the enzyme cannot be revived permanently. Although numerous potential anti-obesity medications have been reported (Ji et al., 2020). Their significant side effects, including vomiting, insomnia, and cardiovascular risks (Cho & Kim, 2020), underscore the need for a safe and natural alternatives for obesity management. Bioactive peptides are considered a potential α-amylase inhibitor (Jahandideh, Bourque, & Wu, 2022). However, the knowledge of its interaction with α-amylase was limited. The calcium (Ca)-binding site is present in the structural domain of α-amylase (Williams, Li, Withers, & Brayer, 2012). Therefore, it is speculated that bioactive peptides that can bind to the Ca-binding site in α-amylase can inhibit α-amylase activity.

In recent years, bioactive peptides derived from seafood have become a hot spot for research (Ozogul et al., 2021), and it was found that the peptides had good calcium binding ability (Cui et al., 2018). Salmon bone is a by-product of salmon processing (Nikoo, Regenstein, & Yasemi, 2023), and which contain a lot of protein (Nikoo, Benjakul, & Ahmadi Gavlighi, 2022) is a good source of Ca-binding peptides. This study aimed to explore its Ca-binding peptides that can inhibit the activity of α-amylase by targeting the active Ca domain in α-amylase. Calcium-binding peptides were extracted from salmon bone, and the obtained peptides exhibit good α-amylase inhibitory activity. The results of this research offer a theoretical and practical foundation for using calcium-binding peptides to selectively inhibit α-amylase during digestion, presenting a promising strategy for obesity prevention and the development of functional foods.

## 2 Materials and methods

### 2.1 Materials and chemicals

Pacific salmon bones were obtained from Dalian Rich Foods Co., Ltd. (Liaoning, China). The peptide IEELEEELEAER(PIE), with a purity of 94.74% was synthesized at ChinaPeptides CO., Ltd. (QYAOBIO) in Shanghai, China. α-Amylase was obtained from Shanghai Yuanye Biotechnology Co., Ltd. (Shanghai, China). Penicillin–streptomycin (PS) and fetal bovine serum (FBS) were purchased from GIBCO (New York, USA). Formic acid was of mass spectrometry grade. Liquid chromatography-grade acetonitrile (ACN) was bought from Merck (Darmstadt, Germany).

### 2.2 Preparation and characterization of salmon bone hydrolysates

#### 2.2.1 Preparation of salmon bone hydrolysates

The fish bones were autocla*v*ed at 121 °C for 40 min, followed by dehydration at 75 °C for 48 h. The dried bones were subsequently grounded into fine powder using a pul*v*erizer (BLF-YB2000, China). Fish bone powder was mixed with 5% NaCl solution at a ratio of 1:5 (*w*/*v*) and the mixture were magnetically stirred at 25 °C for 6 h to remove tramp proteins. Subsequently, the samples were filtrated, washed, and dried at 75 °C. The washed fishbone powder was mixed with deionized water at a ratio of 1:5 (w/v), followed by enzymatic hydrolysis step, using pepsin, trypsin, and neutral protease respectively. Pepsin and trypsin hydrolysis were conducted at 37 °C for 5 h with 5000 U/g protein, at pH 2.0 and 8.0, respectively. Neutral protease hydrolysis was conducted at 50 °C, pH 7.0, with 5000 U/g protein for 5 h. After enzymatic digestion, the mixtures were neutralized to pH = 7 and heated at 100 °C for 10 min. Following this, the digested solutions were centrifuged at 6000 rpm for 20 min, and the supernatant was collected and stored at −80 °C. The freeze-drying process was performed to prepare salmon bone hydrolysates (Idowu, Benjakul, Sinthusamran, Sookchoo, & Kishimura, 2019). Freeze-drying process was conducted to produce salmon bone hydrolysates, designated as salmon bone pepsin hydrolysate (SBPH), trypsin hydrolysate (SBTH), and neutral protease hydrolysate (SBNPH).

#### 2.2.2 Preparation of Ca-bound hydrolysates complexes

The Ca-binding capacities of SBPH, SBTH, and SBNPH were determined according to the previous study (Choi, Jung, Choi, Kim, & Ha, 2005). 5 mg lyophilized powder of SBPH, SBTH, and SBNPH, was mixed with 1 mL of 5 mM CaCl_2_ and 2 mL of phosphate-buffered saline (PBS, 20 mM, pH = 7). The mixture was stirred at 37 °C pH = 7 for 1 h, followed by centrifugation at 5000 rpm for 10 min. The calcium content in the supernatant was determined using colorimetry with Ca assay kits (JianCheng, Nanjing, China).

The SBPH-Ca, SBTH-Ca, and SBNPH-Ca complexes were prepared according to a previously reported study (Cui et al., 2018). SBPH, SBTH, and SBNPH were accurately weighed and dissolved in ultrapure water. CaCl_2_ was then mixed with peptides in a ratio of 3:1 (*w*/w) at 50 °C and pH 8.0. Thereafter, the reactive solution was stirred continuously for 1 h. During the reaction, the pH of the reaction solution was adjusted to 8.0 to prepare SBPH-Ca, SBTH-Ca, and SBNPH-Ca complexes. Anhydrous ethanol was added to the reaction system to reach a final concentration of 90%. The SBPH-Ca, SBTH-Ca, and SBNPH-Ca complexes were precipitated after a standing time of 60 min. Free Ca was then separated by centrifugation at 10,000 rpm for 10 min, and the sediment was recovered and lyophilized to obtain SBPH-Ca, SBTH-Ca, and SBNPH-Ca.

#### 2.2.3 Free amino acid composition of salmon bone hydrolysates and hydrolysate-Ca complexes

Free amino acid composition in SBPH, SBTH, SBNPH, SBPH-Ca, SBTH-Ca, and SBNPH-Ca was measured using a fully automated amino acid analyzer (L-8900, Hitachi, Japan). The 5 mg/mL samples were prepared and mixed with acetone in a ratio of 1:3 to stand for 60 min. Subsequently, the samples were centrifuged at 10,000 rpm for 5 min, and the resulting reaction solutions were subjected to rotary evaporation, followed by being redissolved with 0.02 M HCl. Finally, the samples were filtered through a 0.45-μm microporous membrane and placed in the Hitachi L-8900 amino acid analyzer for determination by post-column ninhydrin derivatization photometry (Zhu, Zhang, Kang, Zhou, & Xu, 2014).

#### 2.2.4 Molecular weight distribution of salmon bone hydrolysates

The molecular weight distribution range of SBPH, SBTH, and SBNPH was determined using the Elite-P230 semi-preparati*v*e high-performance liquid chromatography (HPLC) system with a gel column (Superdex Peptide 10/300 GL, 300 mm × 10 mm). The lyophilized powders of SBPH, SBTH, and SBNPH were dissolved in ultrapure water to prepare solutions with a concentration of 1 mg/mL. After filtering through a 0.45-μm microporous membrane, the samples were loaded onto the HPLC system (mobile phase: ACN/water = 40:60, *v*/v). A sample volume of 10 μL was injected into the system, and the elution was carried out at a flow rate of 0.4 mL/min. Detection of eluted compounds was performed at a wavelength of 220 nm. To determine the molecular weight distribution, molecular weight standards were employed, including glycine (75 Da), reduced glutathione (307 Da), vitamin B12 (1355 Da), β-amyloid (4514 Da), peptidase (6512 Da), and cytochrome C (12,500 Da) (Liu, Lv, Xu, & Guo, 2016).

#### 2.2.5 Spectroscopic characterization of salmon bone hydrolysates

The lyophilized powders of SBPH, SBTH, and SBNPH were dissolved in ultrapure water followed by configured to get 0.1 mg/mL solutions. Various concentrations of CaCl_2_ were then added to achieve final concentrations of 0, 50, 100, 500, and 1000 mM in the reaction solution. The reactions were conducted at pH 8.5 and 50 °C for 60 min. Subsequently, the reacted samples were scanned by a UV–visible (Vis) spectrometer from 200 to 800 nm with the speed of 1200 nm/min(Huang et al., 2021).

For fluorescence spectrometry, the samples were prepared using the same procedure as described above. The reacted samples were scanned using a fluorescence spectrometer (Hitachi Co., Tokyo, Japan), with a scanning range of 310–500 nm and a slit width of 10 nm (Bao, Zhang, Sun, & Lin, 2021).

To conduct fourier transform infrared (FTIR) spectrometry, 2 mg lyophilized SBPH, SBTH, and SBNPH powders were mixed with KBr (200 mg) and grounded. The samples were subjected to FTIR spectrometry, with KBr as a blank background. Infrared data were scanned in the 4000–400 cm^−1^ wavenumber range (Huang et al., 2021).

The secondary structures of SBPH, SBTH, SBNPH, SBPH-Ca, SBTH-Ca, and SBNPH-Ca were obtained using the Jasco J-810 circular dichroism (CD) spectrometer (Jasco Co., Tokyo, Japan) according to a previously reported method (Pal et al., 2021). The concentrations of SBPH, SBTH, SBNPH, SBPH-Ca, SBTH-Ca, and SBNPH-Ca were set at 0.5 mg/mL to match the test voltage. The scan speed was 20 nm/min, the scan range was set to 190–260 nm, the slit width was 0.2 nm, and the cuvette optical range was 1 mm. The experiment was performed in triplicates for each sample.

#### 2.2.6 Particle size characterization of salmon bone hydrolysates

The average particle sizes of the lyophilized SBPH, SBTH, SBNPH, SBPH-Ca, SBTH-Ca, and SBNPH-Ca powders were determined using a dynamic light scattering particle size meter (SZ-100, HORIBA, Japan. To conduct the measurements, the samples were dissolved in ultrapure water at a concentration of 1 mg/mL. Each sample was tested at 25 °C, and measurements were performed in triplicate (Cui et al., 2019).

#### 2.2.7 Identification of salmon bone trypsin hydrolysate

Experimental mass spectrometry data were retrieved using Maxquant (v1.6.15.0). Inverse libraries were added to the parameter settings to calculate the false positive rate due to random matches. Common contamination libraries were also added to eliminate the effect of contaminating proteins in the identification results. The FDR was set to 1% for both PSM identification and protein identification. Cysteine alkylation C was set as a fixed modification. Variable modifications were oxidation of methionine and acetylation of the N-terminus of the protein. The mass error tolerance for primary parent ions was set to 2.0 ppm for First search and 4.5 ppm for Main search. The secondary fragmentation ions were set to 20 ppm. The maximum number of peptide modifications was 5. The minimum length was 7 amino acid residues. The number of missed cleavage sites was 2, and the enzyme digestion mode was Trypsin/P. The screened peptides were characterized by HPLC as well as ESI-MS (Yu et al., 2017).

### 2.3 Inhibitory effects of salmon bone hydrolysates and PIE on α-amylase activity

#### 2.3.1 Determination of the inhibitory effects of salmon bone hydrolysates on α-amylase activity

The activity of α-amylase was determined spectrophotometrically using soluble starch as the substrate and 3,5-dinitrosalicylic acid (DNS) as the chromogenic agent. α-amylase solution (4 units/mL, 0.25 mL) and 0.25 mL each of SBPH, SBTH, and SBNPH (each at 1 μg/mL, 10 μg/mL, 100 μg/mL, and 1000 μg/mL concentrations) were added to 0.5 mL of PBS (pH 6.9, 0.2 mol/L) to make test solutions. These solutions were then incubated at 37 °C for 10 min. Then, 0.5 mL of 1% soluble starch solution was added to each test solution to continue the reaction for 5 min. Thereafter, l mL of DNS reagent was added to each solution, and the mixtures were boiled for 10 min. After the dilution of five times, the absorbance value was measured at 540 nm (Wu et al., 2019). The inhibition of the activity of α-amylase by salmon bone hydrolysate was calculated as using the formula (1):

(1) $$I\%=[1-\left(B-b\right)/\left(A-a\right)\times 100\%$$

where *I* represents the inhibition rate of α-amylase by salmon bone hydrolysates. A represents the absorbance value of the blank tube, a represents the absorbance value of the blank control tube, B represents the absorbance value of the inhibition tube, and b represents the absorbance value of the background control tube.

#### 2.3.2 In vitro simulation of gastrointestinal digestion

A gastrointestinal digestion simulation test was performed according to a previously reported method (Brodkorb et al., 2019). Samples were assessed at 0, 10, 30, 60, 90, 120, 130, 150, 180, 210, and 240 min. The inhibition rates of SBPH, SBTH, and SBNPH for α-amylase were determined by the DNS method.

#### 2.3.3 α-amylase conformational studies

The effect of peptides on the secondary structure of α-amylase was analyzed by circular dichroism. α-Amylase was reacted with the following different concentrations of SBPH, SBTH, and SBNPH: 1, 10, 100, and 1000 μg/mL. The test parameters were as follows: 1 mm quartz cuvette; scanning wavelength range = 190– 250 nm; bandwidth = 2 nm; scanning speed = 100 nm/min; and response time = 1 s. α-Amylase without SBPH, SBTH, and SBNPH treatment was used as the blank control, and it was analyzed under the same conditions. The experimental results were obtained by subtracting the buffer background from the scanned data (Zhang & Ma, 2013).

#### 2.3.4 Dynamic light scattering (DLS) analysis of α-amylase

The α-amylase solution (4 units/mL) and SBPH, SBTH, and SBNPH solutions (each at 1, 10, 100, and 1000 μg/mL concentrations) were prepared. Filtration was performed using a 0.45-μm filter. The α-amylase solution was incubated with different concentrations of SBPH, SBTH, and SBNPH solutions for 24 h. Each sample (1 mL) was analyzed using the DLS apparatus. The particle size variation of α-amylase in each solution was analyzed. The α-amylase alone solution was used as a positive control under the same conditions (Xiong et al., 2016).

#### 2.3.5 Kinetics of α-amylase inhibition using SBPH, SBTH, SBNPH and PIE

The kinetics of α-amylase inhibition was performed with minor modifications as previously reported (Cardullo et al., 2020). In the reaction system, 1 mL of soluble starch solution (10 mg/mL) and 1 mL of different concentrations of SBPH, SBTH, and SBNPH (concentrations of 1, 10, 100, and 1000 μg/mL, respectively) were added. a temperature bath was maintained at 37 °C for 30 min. Different concentrations of α-amylase solution were added. The change in absorbance value of the system was determined. Plot the rate of enzymatic reaction versus α-amylase concentration.

To the reaction system, 1 mL of soluble starch solution of different concentrations (0, 5, 10, 15, 20, 25 mg/mL) was added. The effects of different concentrations of SBPH, SBTH and SBNPH on α-amylase activity were determined at 37 °C in a water bath. Finally, the inverse of the concentration of soluble starch solution was used as the horizontal coordinate and plotted with Lineweaver-Burk plots to determine the type of inhibition of α-amylase by SBPH, SBTH and SBNP. The general equation for reversible inhibition is given below:

$$v=\frac{v_{\max}S}{K_{m}\left(1+\frac{i}{K_{\mathrm{ic}}}\right)+\left(1+\frac{i}{K_{\mathrm{iu}}}\right)}$$

$$\frac{1}{v}=\frac{K_{m}+s}{v_{\max}s}+\frac{i\left(\frac{K_{m}}{K_{\mathrm{ic}}}+\frac{s}{K_{\mathrm{iu}}}\right)}{v_{\max}s}$$

$$\frac{1}{v}=\frac{K_{m}}{v_{\max}}\left(1+\frac{i}{K_{\mathrm{ic}}}\right)+\frac{s}{v_{\max}}\left(1+\frac{i}{K_{\mathrm{iu}}}\right)$$

where K_m_ is the Michaelis-menten constand (mg/mL), v denotes the initial reaction rate (U, mm/min), S denotes the substrate concentration (mg/mL), i denotes the content of the inhibitor concentration (U, mg/mL), K_ic_ denotes the inhibitory constant of the competitive inhibitor, and K_iu_ denotes the inhibitory constant of the non-competitive inhibitor.

### 2.4 Molecular docking of PIE with α-amylase

The Discovery Studio 2021 client software (Biovia) was used to generate the initial structures of PIE. The docking reactions of PIE with α-amylase were subsequently studied using AutoDock 4.2. The model structure of the PIE–α-amylase complex was optimized based on the density flooding theory (Cui et al., 2019).

### 2.5 Lipid-lowering and weight-loss activity study

#### 2.5.1 Cell culture and differentiation

With minor modifications based on the method (Lee et al., 2023). 3 T3-L1 Preadipocytes were cultured at 37 °C and 5% CO_2_ until 80% fusion, passaged and differentiated. After induction with inducers for 8d, the cells were then cultured with DMEM containing 1% PS and 10% FBS for 4d.

#### 2.5.2 Cell viability assay

With slight modifications based on the method (Cui et al., 2019). MTT method was used to assess the proliferative effects of different concentrations of orlistat, SBTH and PIE on 3 T3-L1 preadipocytes. Cells were treated with different samples for 24 h. Finally, the absorbance at 490 nm was measured using a Microplate Reader (Synergy H1, BioTek, USA).

#### 2.5.3 Measurement of triglyceride content

Cellular triglyceride content (TG) was measured using a kit (JianCheng, Nanjing, China). Differentiated adipocytes were treated with different samples in 6-well plates according to the manufacturer's instructions. Cells were first washed with PBS, followed by the addition of ethanol. The cells were then transferred into centrifuge tubes using a cell scraper. Subsequently, the cells were broken down by ultrasound in an ice water bath. Total cholesterol content was measured using a specialized kit.

#### 2.5.4 Measurement of total cholesterol content

Total cellular cholesterol content was measured using a kit (JianCheng, Nanjing, China). The GPO-PAP enzymatic method was used according to the manufacturer's instructions. Protein concentration was measured using the BCA protein assay kit (Solarbio, Beijing, China).

#### 2.5.5 Determination of lipid content

Lipid content was measured according to previous with minor modifications (Lee et al., 2023). Differentiated cells were washed 3 times with PBS and subsequently fixed with formaldehyde. After fixation, the samples underwent three additional washes with ultrapure water and were then treated with isopropanol for 5 min. Subsequently, oil red O was added for staining. After staining, the dye was eluted with 100% isopropanol, and the absorbance at 500 nm was measured.

#### 2.5.6 Effect of PIE peptide on body weight in mice

The animal study was approved by the Animal Ethics Committee of Dalian Polytechnic University (protocol ID SYXK2017–0005) per the guidelines of the National Institutes of Health Laboratory Animals for Animal Care. 10-week-old female BALB/c mice (L20160258, Dalian Medical University, Liaoning, China) were acclimatized with normal chow for 2 d, and were randomly separated 2 groups (*n* = 8): the control group (saline) and the PIE group (at a dose of 30 mg PIE/kg body weight). Both groups were maintained on a basal diet throughout the study. Given that mice are nocturnal feeders, gavage was performed between 5:00–6:00 PM. Body weights were measured and recorded at 7-day intervals. The feed and water were provided ad libitum to mice. Changes in body weight were analyzed after 12 weeks of continuous gavage (Pinzón-García et al., 2018).

### 2.6 Statistical analyses

The results are expressed as the mean ± standard deviation. International Business Machines Statistical Package for Social Sciences 26.0 was used for statistical analyses of the data. Data were analyzed by Duncan's post-hoc test. The *P*-value <0.05 was considered statistically significant.

## 3 Results and discussion

### 3.1 Preparation and characterization of Ca-binding hydrolysate from salmon bone

**Fig. 1:**
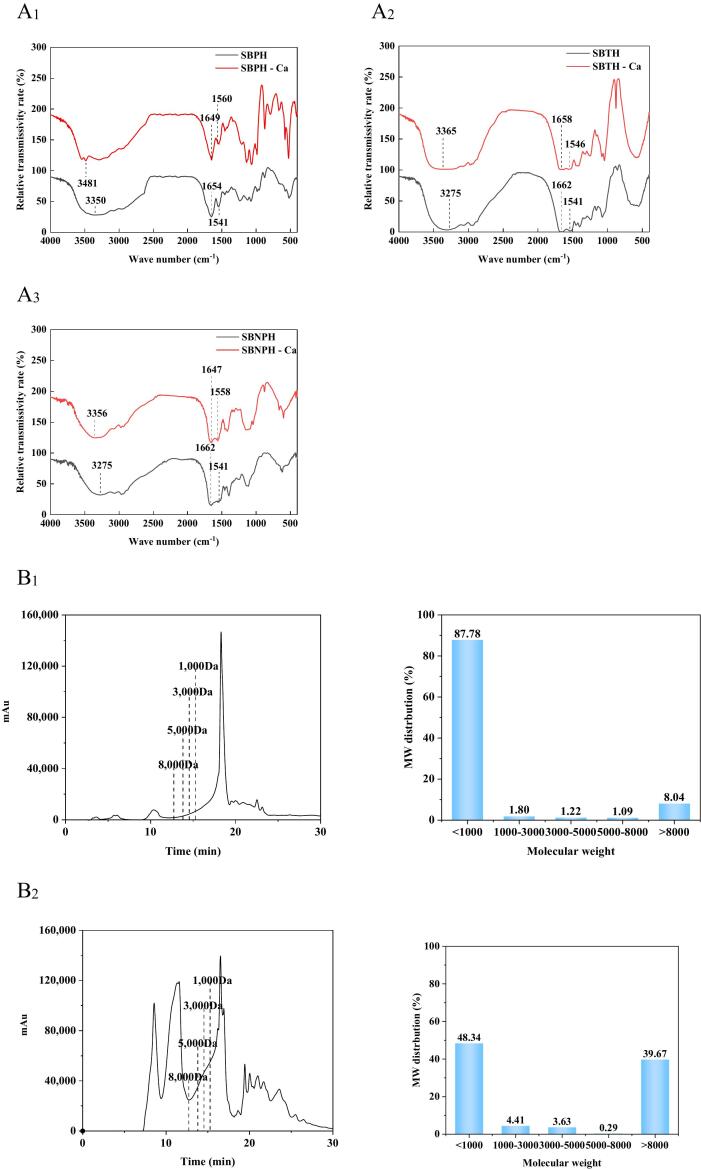

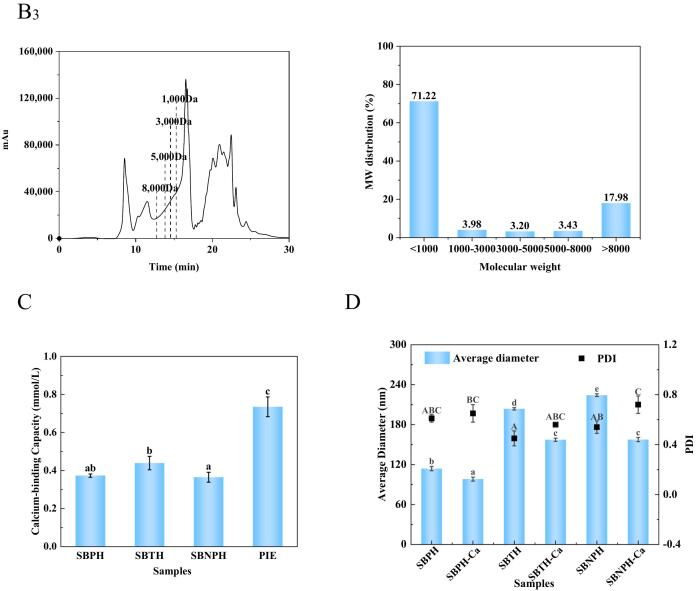
Preparation and Characterization of Calcium (Ca)-binding Hydrolysate from Salmon Bone. A_1–3_: Fourier transform infrared analysis of the hydrolysate and the corresponding hydrolysate-Ca complexes obtained from the hydrolysis of the salmon bone with three enzymes in the range of 4000–400 cm^−1^. (A_1_) salmon bone pepsin hydrolysate (SBPH), (A_2_) salmon bone trypsin hydrolysate (SBTH), and (A_3_) salmon bone neutral protease hydrolysate (SBNPH). B_1–3_: Molecular weight distribution curve and percentage of the salmon bone protease hydrolysate. (B_1_) SBPH, (B_2_) SBTH, and (B_3_) SBNPH. C: Ca-binding capacity of hydrolysates obtained by the enzymatic digestion of the salmon bone by different proteases. D: Particle size variation of salmon bone protein hydrolysates and their complexes. Data were analyzed by Duncan's post-hoc test. Different letters indicate significant differences (*P* < 0.05). All values were the means ± SD (*n* = 3).

The results of the free amino acid composition in SBPH, SBTH, SBNPH, and their calcium-complexed forms were shown in Table 1. The glutamic acid (Glu) contents in SBPH-Ca, SBTH-Ca, and SBNPH-Ca were significantly lower than their non-complexed counterparts (*P* < 0.05), suggesting a preferential binding of Glu-containing peptides to calcium. Additionally, FTIR analysis (Fig. 1A1–3) showed that that the N—H bond vibration peaks for SBPH, SBTH, and SBNPH at 3350 cm^−1^, 3275 cm^−1^, and 3275 cm^−1^, respectively, shifted to higher frequencies upon calcium chelation (3481 cm^−1^, 3365 cm^−1^, and 3356 cm^−1^, respectively). indicating the interaction between the protein hydrolysates and calcium ions (Cui et al., 2023). Moreover, a shift of the amide I band (symmetric and asymmetric structures of -COO-) from lower to higher wave numbers was observed (1649 cm^−1^, 1658 cm^−1^, and 1647 cm^−1^ to 1654 cm^−1^, 1662 cm^−1^, and 1662 cm^−1^, respectively), indicating potential binding of the -COO- group to calcium. These findings align with previous research on wheat germ protein hydrolysate-Ca complexes (Liu et al., 2016), and suggest that SBPH, SBTH, and SBNPH can form complexes with calcium through interactions involving the carboxyl and amino groups of Glu.

**Table 1:** Analysis of free amino acid composition of SBPH, SBTH, SBNPH and SBPH-Ca, SBTH-Ca, SBNPH-Ca.

Amino acids	Relative content (%)					
	SBPH	SBPH-Ca	SBTH	SBTH-Ca	SBNPH	SBNPH-Ca
Asp	2.26 ± 0.04^e^	0.99 ± 0.04^d^	0.43 ± 0.00^b^	0.10 ± 0.01^a^	0.98 ± 0.00^d^	0.73 ± 0.05^c^
Thr	1.16 ± 0.08^e^	0.34 ± 0.01^b^	0.56 ± 0.18^c^	0.07 ± 0.02^a^	1.52 ± 0.03^f^	0.96 ± 0.10^d^
Ser	1.58 ± 0.11^d^	1.27 ± 0.05^c^	0.47 ± 0.16^b^	0.22 ± 0.05^a^	1.60 ± 0.03^d^	1.54 ± 0.08^d^
Glu	2.34 ± 0.16^d^	1.85 ± 0.04^c^	0.97 ± 0.01^b^	0.06 ± 0.01^a^	6.23 ± 0.03^e^	2.35 ± 0.01^d^
Gly	2.81 ± 0.20^d^	3.14 ± 0.04^e^	1.68 ± 0.03^b^	1.86 ± 0.07^b^	1.32 ± 0.13^a^	2.36 ± 0.08^c^
Ala	2.74 ± 0.17^c^	1.15 ± 0.13^ab^	1.43 ± 0.22^b^	0.81 ± 0.04^a^	4.86 ± 0.41^d^	2.38 ± 0.18^c^
Val	2.62 ± 0.00^c^	1.57 ± 0.55^b^	2.01 ± 0.18^b^	0.83 ± 0.22^a^	3.74 ± 0.09^d^	2.83 ± 0.19^c^
Cys	0.53 ± 0.16^a^	1.30 ± 0.83^b^	1.16 ± 0.04^ab^	0.86 ± 0.02^ab^	4.09 ± 0.09^d^	2.87 ± 0.01^c^
Met	1.66 ± 0.42^c^	0.33 ± 0.09^ab^	0.03 ± 0.01^a^	0.02 ± 0.01^a^	2.37 ± 0.08^d^	0.59 ± 0.04^b^
Ile	1.34 ± 0.14^b^	0.04 ± 0.01^a^	0.01 ± 0.00^a^	0.07 ± 0.02^a^	4.65 ± 0.08^c^	1.44 ± 0.18^b^
Leu	3.07 ± 0.21^c^	0.05 ± 0.02^a^	0.11 ± 0.05^a^	0.02 ± 0.00^a^	4.85 ± 0.34^d^	1.56 ± 0.07^b^
Tyr	1.12 ± 0.04^b^	0.40 ± 0.09^a^	1.40 ± 0.06^c^	1.57 ± 0.08^d^	1.46 ± 0.12^cd^	0.54 ± 0.06^a^
Phe	2.48 ± 0.42^bc^	1.40 ± 0.22^ab^	2.02 ± 0.54^bc^	2.55 ± 0.04^c^	2.46 ± 0.11^bc^	0.46 ± 0.14^a^
Lys	0.29 ± 0.03^a^	1.09 ± 0.08^b^	1.01 ± 0.20^b^	4.60 ± 0.25^d^	0.97 ± 0.10^b^	1.67 ± 0.03^c^
His	0.23 ± 0.02^a^	1.37 ± 0.21^c^	1.39 ± 0.07^c^	1.83 ± 0.05^d^	0.53 ± 0.15^b^	1.56 ± 0.11^c^
Arg	0.12 ± 0.04^a^	1.56 ± 0.17^c^	0.88 ± 0.22^b^	3.23 ± 0.49^d^	0.31 ± 0.01^a^	1.64 ± 0.03^c^

Notes: The results are expressed as the mean ± standard error (n = 3), and Different letters in the same row indicate significant differences between samples (*P* < 0.05).

Abbreviation: SBSH, salmon bone pepsin hydrolysate; SBTH, salmon bone trypsin hydrolysate; SBNPH, salmon bone neutral protease hydrolysate.

Herein, the molecular weight distribution of salmon bone hydrolysates was analyzed, and the results are shown in Fig. 1B1–3. The average molecular weights of SBPH, SBTH, and SBNPH were 2638 Da, 10,580 Da, and 5358 Da, respectively. The capacity of these hydrolysates to bind calcium was also examined to screen the most effective protease for generating calcium-binding peptides from salmon bone. SBTH emerged as the hydrolysate with the highest calcium-binding capacity, with the peptide PIE from SBTH showing the greatest affinity for calcium (Fig. 1C).

Furthermore, the interaction between organic ligands and metal ions often leads to changes in the UV–Vis absorption spectrum, including the emergence, shift, or disappearance of absorption peaks (Huang et al., 2021). In this study, the UV–Vis spectra of SBPH, SBTH, and SBNPH exhibited a notable absorption band at 260 nm (Fig. S1 A_1–3_), which was characteristic of the phenylalanine (Phe) content in peptides (Bingtong, Yongliang, & Liping, 2020). As the calcium concentration increased, the Phe peak underwent red-shifting and became less pronounced, which was consistent with the observations from studies on the β-lactoglobulin hydrolysate‑iron complex by (Zhou et al., 2012). The results indicated that after binding with Ca, the chiral spatial structure of peptide chromophores (C=O and -COOH) and the co-color groups (-OH and -NH_2_) on the peptide chain were changed, suggesting that new compounds were generated after Ca interacted with SBPH, SBTH, and SBNPH.

The folding of the peptide can lead to a decrease in fluorescence intensity. The endogenous fluorescence intensity of the sample decreased notably with the increase of CaCl_2_ because of the fluorescence quenching action of Ca (Fig. S1 B_1–3_). The results indicated that the binding of Ca to peptides prompted folding within the peptide structure, causing the indole group of tryptophan to move from an exposed surface location to a more shielded interior position. Compared with SBPH, SBTH, and SBNPH, β-foldings in the SBPH-Ca, SBTH-Ca, and SBNPH-Ca complexes increased, and the irregular helix content decreased (Fig. S1 C_1–3_). This suggests that SBPH-Ca, SBTH-Ca, and SBNPH-Ca complexes may have been formed by the self-assembly behavior of the peptides with β-folded structures. However, during simulated gastrointestinal digestion, there was a notable decrease in β-folding and an increase in irregular helix structures, leading to a diminished capacity of the peptides to bind calcium (Cui et al., 2023).

Particle size is one of the important physical properties of nanocomposites and depends on the volume of the particles (Hu et al., 2022). The average particle size distributions of SBPH, SBTH, SBNPH, SBPH-Ca, SBTH-Ca, and SBNPH-Ca, as measured by DLS, are shown in Fig. 1D. The average particle sizes of SBPH, SBTH, SBNPH, SBPH-Ca, SBTH-Ca, and SBNPH-Ca were 113.97 nm, 203.73 nm, 224.23 nm, 98.2 nm, 157.3 nm, and 157.57 nm, respectively. Notably, the particle sizes for the calcium-bound forms were smaller than those of their unbound counterparts. This reduction in size is attributed to the structural folding that occurs during the chelation process, where the peptides bind with calcium ions to form more compact nanoparticles. It was also demonstrated that the presence of Ca led to the folding of the peptides, resulting in the formation of whey hydrolysate-Ca complexes (Huang et al., 2021).

### 3.2 Inhibitory effects of salmon bone hydrolysates on α-amylase activity

**Fig. 2:**
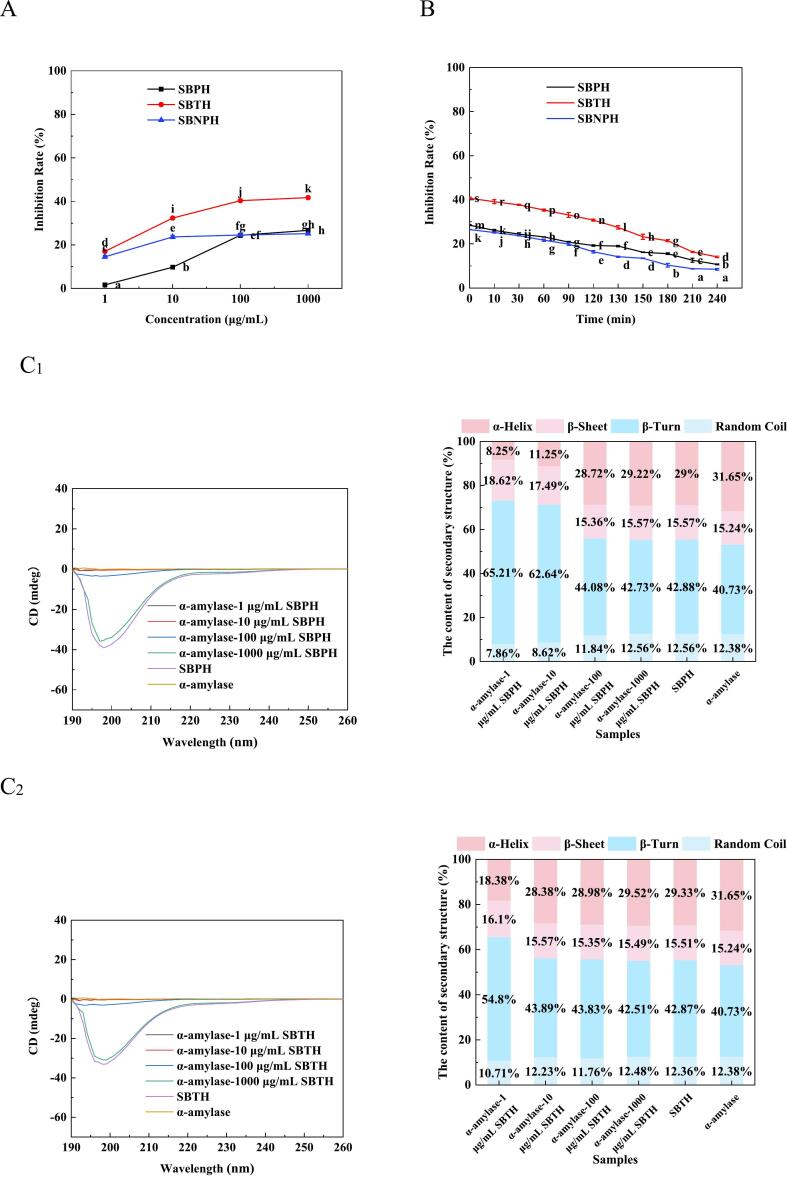

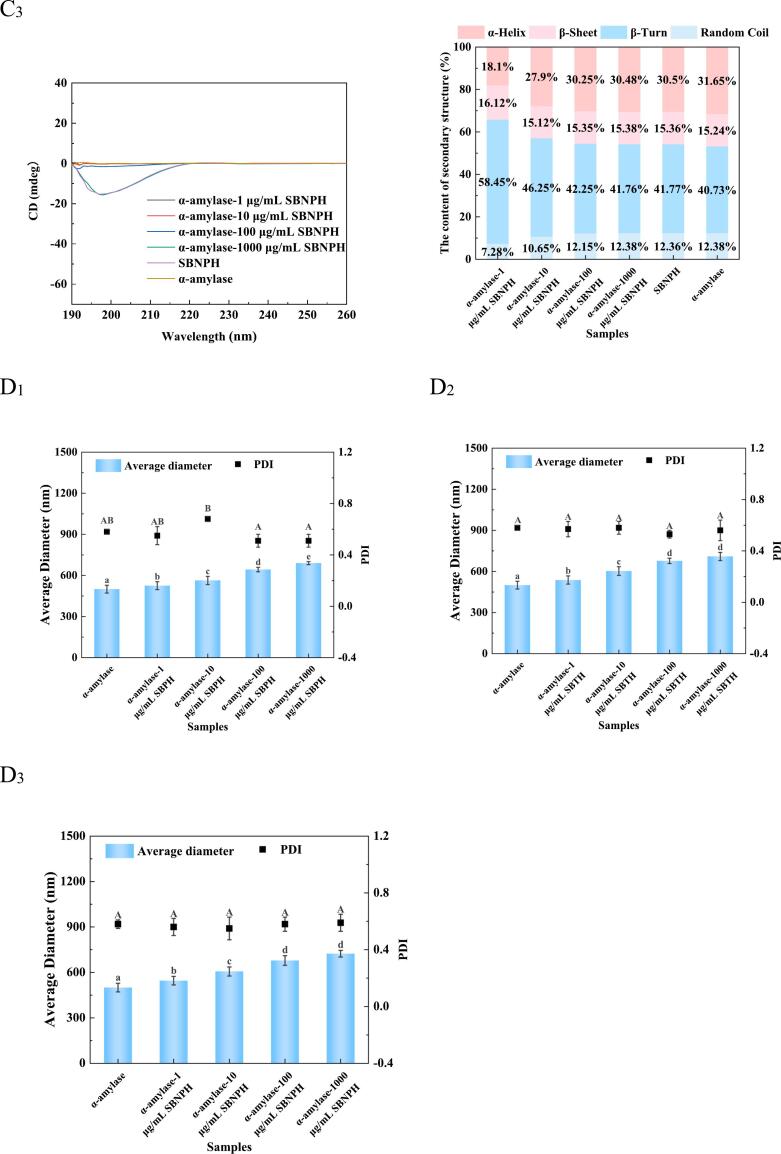
Effect of the Salmon Bone Hydrolysate on α-Amylase Inhibitory Activity. A: The effect of the inhibition rate with salmon bone pepsin hydrolysate (SBPH), salmon bone trypsin hydrolysate (SBTH), and salmon bone neutral protease hydrolysate (SBNPH). B: Effect of the inhibition rate with simulated digestion time in vitro. C_1–3_: Effect of different protease hydrolysate concentrations on the secondary structure content of α-amylase. (C_1_) SBPH, (C_2_) SBTH, and (C_3_) SBNPH. D_1–3_: Effect of peptide concentrations of different protease hydrolysates on α-amylase particle size (D_1_) SBPH, (D_2_) SBTH, and (D_3_) SBNPH. Data were analyzed by Duncan's post-hoc test. Different letters indicate significant differences (*P* < 0.05). All values were the means ± SD (n = 3).

α-Amylase, as primary enzyme required for starch hydrolysis, catalyzes the hydrolysis of the α-1,4 glycosidic bond in starch. Its inhibition plays a crucial role in moderating postprandial blood glucose levels by curtailing the absorption of simple sugars (Guzmán, Garza, Ríos, Minsky, & Aranda, 2022). Consequently, α-amylase inhibitors could be applied in controlling post-meal hyperglycemia. In the comparative analysis at a concentration of 100 μg/mL, SBTH, SBPH, and SBNPH showed α-amylase inhibition rates of 40.35%, 24.44%, and 24.56%, respectively, with SBTH rendering the most effective inhibition (Fig. 2A). This efficacy parallels the high Ca-binding capacity observed in SBTH, indicating a correlation between Ca-binding and α-amylase inhibitory potential (Sandate-Flores et al., 2022). However, during a simulation of gastrointestinal digestion, a decline in the inhibition rate was observed (Fig. 2B), attributable to gastrointestinal proteases disrupting the peptide sequences responsible for α-amylase inhibition (Montiel-Aguilar et al., 2020).

The CD spectrogram of α-amylase changed notably after the addition of SBPH, SBTH, and SBNPH, indicating the exist of structural changes (Fig. 2C1–3). Specifically, the α-helix content decreased while the β-helix content increased continuously with the increase concentration of the hydrolysates, suggesting that these peptides can induce the conformational change of α-amylase and reduce its active site. Thus, the activity of α-amylase was inhibited. Furthermore, the hydrodynamic radius of α-amylase increased with the addition of SBPH, SBTH, and SBNPH, starting from a baseline of 500.2 nm. (Fig. 2D1–3). This suggests that the peptides served as nuclei for the aggregation of α-amylase, altering the enzyme's aggregation and leading to larger copolymer formations (Lu et al., 2017b).

**Fig. 3:**
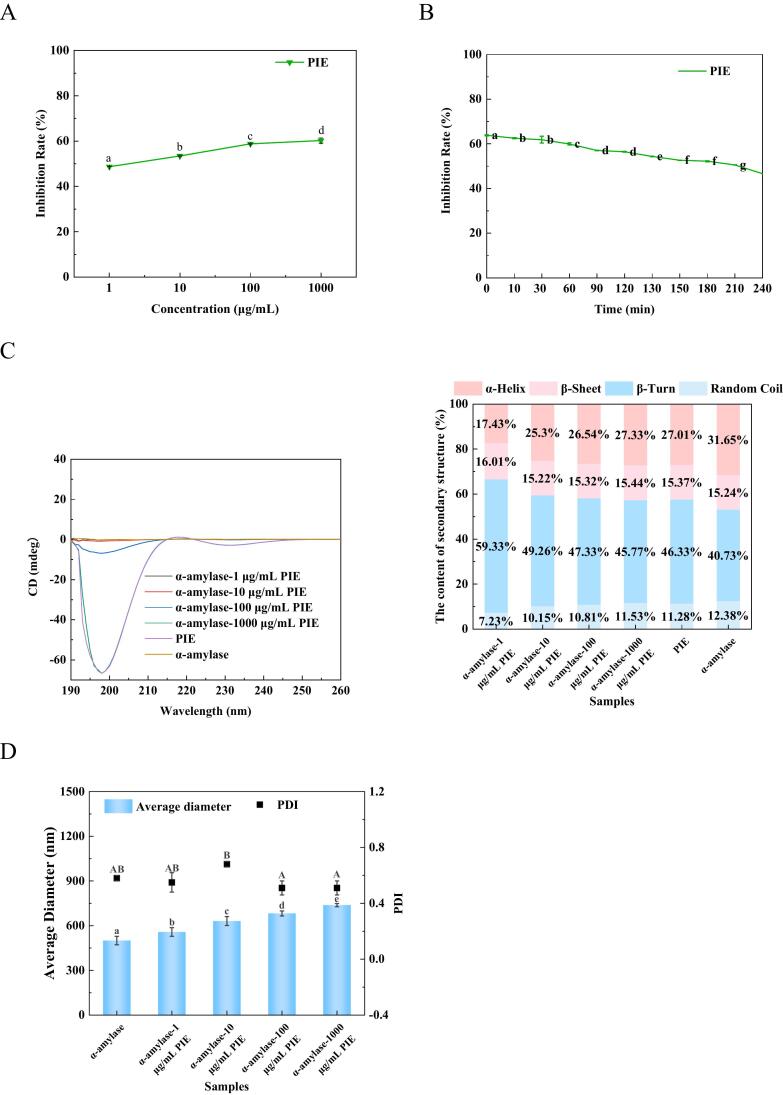
Effect of PIE on α-Amylase Inhibitory Activity. A: Effect of the inhibition rate with PIE. B: Effect of the inhibition rate with simulated digestion time in vitro. C: Effect of PIE on the secondary structure content of α-amylase. D: Effect of concentrations of PIE on α-amylase particle size. Data were analyzed by Duncan's post-hoc test. Different letters indicate significant differences (*P* < 0.05). All values were the means ± SD (n = 3).

As shown in Fig. S2(A_1_-A_3_), the mode of inhibition of α-amylase by SBPH, SBTH, and SBNPH was explored. As can be seen from the Figure, SBPH, SBTH, and SBNPH had the same characteristics: graphing the α-amylase concentration against the enzyme reaction rate showed that all the straight lines in SBPH, SBTH, and SBNPH intersected and intersected at the origin, and their slopes were decreasing with the increase of the concentration of SBPH, SBTH, and SBNPH, which indicated that SBPH, SBTH, SBNPH all have reversible inhibition process on α-amylase. The decrease of the slope of SBTH was larger than that of SBPH and SBNPH, and the straight line of the SBTH group is closer to the horizontal coordinate when SBPH, SBTH, and SBNPH all reach the highest concentration. Therefore, it was shown that SBTH had a stronger inhibitory effect on α-amylase compared to SBPH and SBNPH (Lu et al., 2017a).

Enzyme inhibition is classified into four types—mixed, anticompetitive, non-competitive, and competitive—judged on the Lineweaver-Burk plot. The type of inhibition can be discerned by the intersection point of the lines on this plot. Mixed inhibition is indicated when lines intersect in the second or third quadrant. Anticompetitive inhibition is characterized by parallel lines, non-competitive inhibition by lines intersecting the x-axis, and competitive inhibition by lines intersecting the y-axis (Fenoll, García-Ruiz, Varón, & García-Cánovas, 2003; Oyama et al., 2016).

Fig. S2(B_1_-B_3_) revealed that the lines for SBPH, SBTH, and SBNPH intersect in the second quadrant, signifying mixed inhibition. This indicates that these hydrolysates can bind to α-amylase at sites distinct from the active site, showing non-competitive inhibition, as well as at the active site itself, exerting competitive inhibition. The inhibition constants K_I_ and K_IS_ of SBPH, SBTH, and SBNPH on α-amylase were calculated by Lineweaver-Burk double inverse plots. As shown in Table S2, the K_IS_ values of SBPH, SBTH, and SBNPH on α-amylase were greater than the K_I_ values, while smaller inhibition constants indicate stronger enzyme-compound ability and better inhibition of enzyme (Jiang et al., 2021). SBTH showed the smallest K_I_ and K_IS_ among the hydrolysates, confirming its superior inhibitory action on α-amylase

### 3.3 Effect of PIE on α-amylase inhibitory activity

Peptidomics facilitates the comprehensive characterization of peptides on a large scale. This analytical approach offers a time-efficient means of generating an extensive array of peptide fragments (Martini, Solieri, & Tagliazucchi, 2021). Herein, the peptide sequences were retrieved from an established database using MASCOT, and active peptide sequences with Ca-binding capacities were identified. The peptide sequences from SBTH were identified in Table S1 (Supplementary materials), along with information on their amino acid sequences, lengths, and molecular weights. A total of 6505 peptide sequences were identified, with *m*/*z* ratios spanning from 400 to 1191. Ca was mainly bound to amino nitrogen and carboxyl oxygen atoms in glutamic acid in SBTH. Peptide IEELEEELEAER (PIE) from SBTH is the most glutamate-containing peptide. PIE not only exhibits robust activity and stability within the gastrointestinal tract but also demonstrates a pronounced calcium-binding capability (Xu et al., 2020; Xu et al., 2019; Xu et al., 2022). Therefore, combined with the above experimental results, peptide PIE may be a potential α-amylase inhibitor.

At a concentration of 100 μg/mL, PIE inhibited α-amylase by 58.81%, outperforming SBPH, SBTH, and SBNPH as depicted in Fig. 3A. This efficacy was attributed to its superior Ca-binding capacity, indicating that PIE may be the main component of SBTH to inhibit α-amylase activity. However, the inhibition rate decreased significantly with the increasing duration of gastrointestinal digestion (Fig. 3B). This may be attributed to the proteolytic breakdown of key peptide sequences in PIE responsible for the α-amylase inhibition (Guzmán et al., 2022).

PIE impact on α-amylase was further substantiated by changes in the enzyme's CD spectrogram (Fig. 3C). An increase in PIE concentration led to a decrease in α-helix content and an increase in β-fold structures, signifying that PIE induced conformational changes in α-amylase, reducing its active site availability, and compromising its enzymatic function (Lu et al., 2017b). PIE addition significantly affected the hydrodynamic radius of α-amylase (Fig. 3D). As PIE concentration increased, the hydrodynamic radius of α-amylase increased gradually. This reason may be PIE is considered the nucleus of the α-amylase aggregate, which allows the aggregation of larger copolymers on top of the original smaller aggregates. PIE addition changed the aggregation mechanism of α-amylase probably by binding to the Ca domain in α-amylase.

As shown in Fig. S3(A), the mode of inhibition of α-amylase by PIE was experimentally investigated. Plotting the enzyme reaction rate against the concentration of α-amylase showed that all the straight lines in PIE intersected and intersected at the origin, and their slopes were decreasing with the increase of the PIE concentration, which indicated that the inhibition of α-amylase by PIE was reversible. The decrease of the slope of PIE was larger than that of SBPH, SBTH, SBNPH. Therefore, the study showed that PIE inhibited α-amylase more strongly compared to SBPH, SBTH, SBNPH (Lu et al., 2017a). Fig. S3 (B) shows that the straight line of PIE intersects the y-axis. Therefore, it can be judged that the type of inhibition of α-amylase by PIE is competitive. The inhibition constants K_I_ and K_IS_ of PIE on α-amylase were calculated by Lineweaver-Burk double inverse plots. As shown in Table S3, the K_IS_ values of PIE on α-amylase were all greater than the K_I_ values, whereas smaller inhibition constants indicate stronger enzyme-compound ability and better inhibition of the enzyme (Jiang et al., 2021). The inhibition constants of PIE on α-amylase, K_I_ and K_IS_, were all smaller than the inhibition constants of SBPH, SBTH, SBNPH on α-amylase. So PIE inhibited α-amylase more.

### 3.4 Targeted inhibition of peptide PIE on α-amylase via molecular docking

Molecular docking techniques are widely used to investigate the mode of ligand–receptor binding (Caballero, 2021). The CDOCKER docking algorithm of the Discovery Studio 2021 software was used in the present study. Based on a schematic representation of molecular dynamics under the CHARMm force field and the receptor–ligand interaction scoring function, interactions between α-amylase and bioactive peptides were successfully evaluated.

**Fig. 4:**
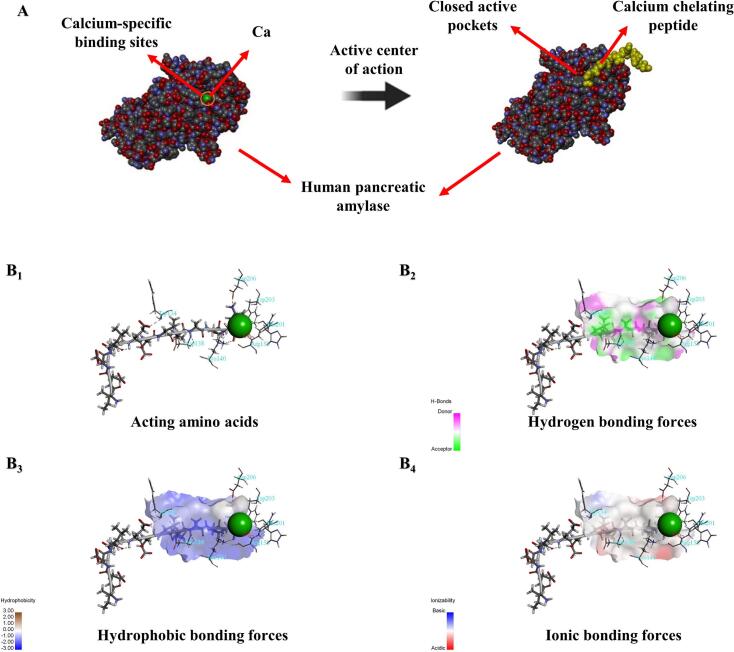
Targeted Inhibition of PIE on α-amylase via Molecular docking. A: Mechanism of α-amylase inhibition by PIE. B: Docking calculations for the interaction of PIE with α- amylase. B_1_: Interaction sites of PIE and amino acids in α- amylase. B_2–4_: The forces between receptors and ligands are hydrogen bonding forces, hydrophobic bonding forces, and ionic bonding forces.

The structure of α-amylase was shown to include calcium ions, which are known to be crucial for the enzyme's stability and catalytic function (Fig. 4A). The addition of PIE, which possesses the Ca-binding ability, covers the active site by binding to the Ca residue in α-amylase. This binding mode effectively renders the calcium ion inactive, thereby inhibiting the enzymatic activity of α-amylase. Crucially, the binding site of PIE encompasses the calcium-binding site of α-amylase, corroborating the results obtained from both the calcium-binding capacity and α-amylase inhibition assays.

The specific amino acids involved in the interaction of PIE with α-amylase were identified as Asp206, Trp203, His201, Arg158, Trp134, Asp138, and Lys140, as illustrated in Fig. 4B_1_. Additionally, non-covalent interactions between the receptor and ligand like hydrogen bonding, hydrophobic bonding, and ionic bonding forces, were also shown in Fig. B_2–4_. A positive correlation was observed between the calcium-binding capacities of the hydrolysates and their inhibitory effects on α-amylase (Fig. 1C) further indicating that PIE's inhibition of α-amylase is mediated through chelation with calcium ions.

### 3.5 Lipid-lowering and weight-loss activity study

**Fig. 5:**
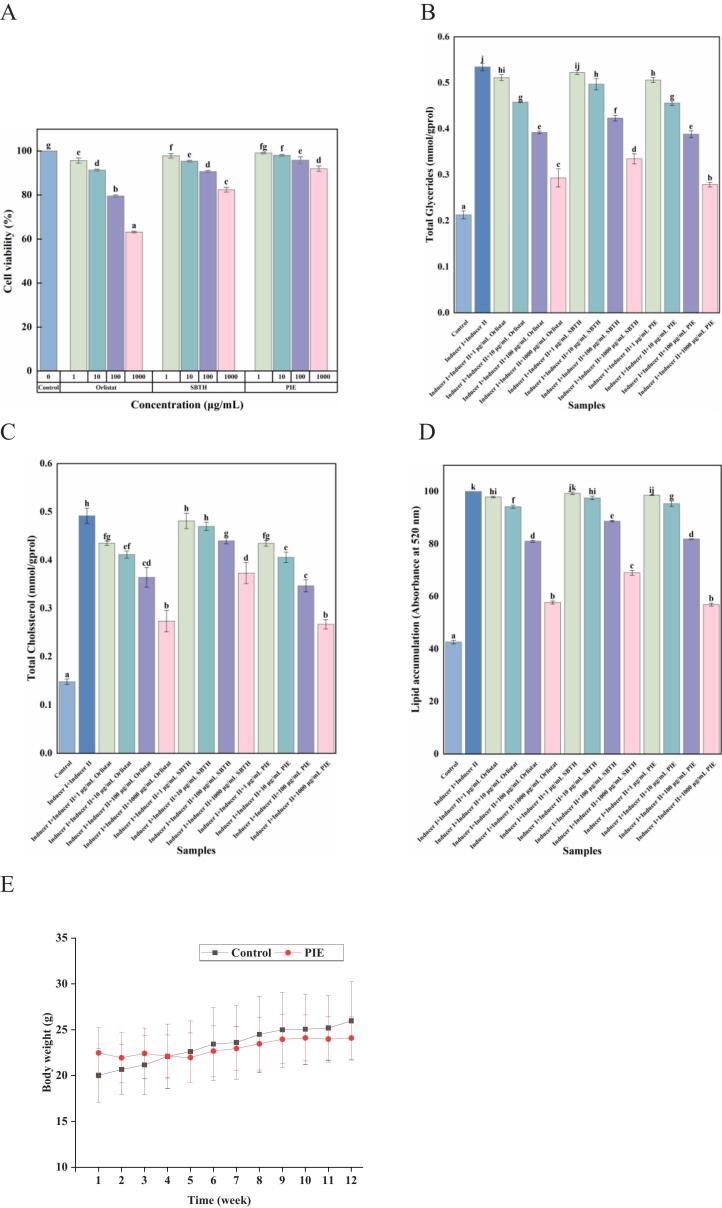
Lipid-lowering and Weight-loss Activity Study. A: Effect of different concentrations of samples on cell viability. B: Effect of different sample concentrations on triglyceride content. C: Effect of different sample concentrations on total cholesterol content. D: Effect of different sample concentrations on lipid content. E: Effect of PIE on body weight in vivo (*n* = 8).

The evaluation of lipid accumulation is commonly based on measuring TG content, total cholesterol (TC), and lipid content (Cheng et al., 2020). According to the results displayed in Fig. 5A, orlistat, SBTH, and PIE showed no cytotoxicity at concentrations ranging from 1 to 100 μg/mL.. As can be seen in Fig. 5B, the TG content in the cells of the control group was 0.21 mmol/gprol, while the addition of Inducer I containing 0.5 μmol/mL 3-isobutyl-1-methylxanthine, 1 μmol/L dexamethasone, and 10 μg/mL insulin, and the inducer containing 10 μg/mL insulin culture solution II (Inducer II) cells elevated TG content to 0.54 mmol/gprol.

However, treatment with 1000 μg/mL SBTH and PIE, in conjunction with Inducers I and II, led to reductions in TG content to 62.60% and 52.11%, respectively In Fig. 5C, the addition of 1000 μg/mL PIE resulted in a significant decrease in TC content to 54.35%, which suggests a strong inhibitory effect on adipocyte differentiation. Similarly, the Fig. 5D reveals a marked inhibition of lipid accumulation. Treatments with orlistat, SBTH, and PIE, in conjunction with Inducers I and II, led to 57.74%, 69.06%, and 56.94% inhibition, respectively. These findings suggest that, while cells could still differentiate and mature into adipocytes, the presence of orlistat, SBTH, and PIE impeded this process. Notably, PIE demonstrated a considerable reduction in TG, TC, and lipid content, paralleling orlistat's effects, hence potentially offering a robust method for inhibiting adipocyte differentiation.

Inhibition digestive enzyme activities, like that of α-amylase, is a strategic approach for managing body weight. This is evident from the 12-week study where gavage of α-amylase inhibitor (peptide PIE) was observed to affect the body weight of mice. After this period, the control group exhibited a normal increase in weight, while the group receiving the peptide PIE showed no significant weight change, as illustrated in Fig. 5. Notably, the weight gain over three months reduced drastically from 33.65% in the control group to a mere 6.61% in the peptide group, assessed from week 1 to week 12. This pronounced decrease in weight gain is attributed to PIE's potent inhibition of α-amylase, facilitated by its strong calcium-binding capacity. The ability of PIE to modulate α-amylase activity suggests its potential application in weight management and obesity prevention by impacting the enzyme's efficiency in digestion.

## 4 Conclusion

The inhibition of digestive enzymes by foodborne molecules are one of the safe and effective ways to reduce for obesity prevention. SBTH exhibited the superior Ca-binding ability and α-amylase inhibition activity. Specifically, the PIE derived from SBTH showed the remarkable α-amylase inhibition rate of 58.81% at a concentration of 100 μg/mL. The activity of α-amylase was inhibited by the binding of PIE to amino acids such as Asp206, Trp203, His201, Arg158, Trp134, Asp138, and Lys140, which were interaction sites for the Ca-active domain in α-amylase. The results of cellular experiments showed that PIE could significantly reduce the total cholesterol content, triglyceride content and lipid content in 3 T3-L1 preadipocytes and effectively inhibit adipocyte differentiation. PIE decreased significantly the body weight gain from 33.65% to 6.61% in the mice study. In conclusion, PIE is a promising molecule for reducing the risk of obesity.

## CRediT authorship contribution statement

**Zhe Xu:** Writing – review & editing, Software, Project administration, Methodology, Funding acquisition, Formal analysis, Data curation. **Shiying Han:** Writing – original draft, Methodology, Formal analysis, Data curation. **Na Cui:** Writing – review & editing, Software. **Hanxiong Liu:** Software, Methodology, Formal analysis, Data curation. **Xu Yan:** Writing – original draft, Formal analysis, Data curation. **Hongrui Chen:** Writing – review & editing, Formal analysis. **Jianping Wu:** Writing – review & editing. **Zhijian Tan:** Project administration, Writing – review & editing. **Ming Du:** Methodology, Writing – review & editing. **Tingting Li:** Funding acquisition, Writing – review & editing.

## Declaration of competing interest

All authors declare that they have no conflicts of interest.

## Data Availability

Data will be made available on request.

## Appendix A Supplementary data

Supplementary material 1 Image 1

Supplementary material 2 **Table S1.** Identification of peptides from SBTH Image 2
