# Supplementary material for: Identification and characterization of a calcium-binding peptide from salmon bone for the targeted inhibition of α-amylase in digestion
Source: Food Chem X. 2024 Apr 3;22:101352. doi: 10.1016/j.fochx.2024.101352 (PMC11004067; doi:10.1016/j.fochx.2024.101352)
Supplement: Supplementary file 1 — Supplementary material 1 [file mmc1.docx]

**Figure Captions**

**Figure S1.** Preparation and Characterization of Calcium (Ca)-binding Hydrolysate from Salmon Bone. **A_1–3_**: Ultraviolet absorption spectra analysis of hydrolysates and corresponding hydrolysate-Ca complexes obtained by the hydrolysis of salmon bones with three enzymes in the 190–800 nm range. **(A_1_)** SBPH, **(A_2_)** SBTH, and **(A_3_)** SBNPH. **B_1–3_**: Fluorescence spectra of the hydrolysates obtained from the hydrolysis of the salmon bone by three enzymes and different concentrations of CaCl_2_ at an excitation wavelength of 295 nm and an emission wavelength range of 310–500 nm **(B_1_)** SBPH, **(B_2_)** SBTH, and **(B_3_)** SBNPH. **C_1–3_**: Circular dichroism spectra and secondary structure content of salmon bone protease hydrolysates and their Ca complexes and their simulated gastrointestinal digestive processes. **(C_1_)** SBPH, **(C_2_)** SBTH, and **(C_3_)** SBNPH.

**Figure S2.** Analysis of α-amylase inhibition by SBPH, SBTH, SBNPH (A) Mode of inhibition (B) Type of inhibition.

**Figure S3.** Analysis of PIE inhibition of α-amylase (A) Mode of inhibition (B) Type of inhibition.

**Table Captions**

**Table S1.** Identification of peptides from SBTH

**Table S2.** Inhibition constants and type of inhibition of α-amylase by SBPH, SBTH, SBNPH.

**Table S3.** Inhibition constants and type of inhibition of α-amylase by PIE

**Figure S1**

A_1_ A_2_

A_3_ B_1_

B_2_ B_3_

C_1_

C_2_

C_3_

**Figure S2**


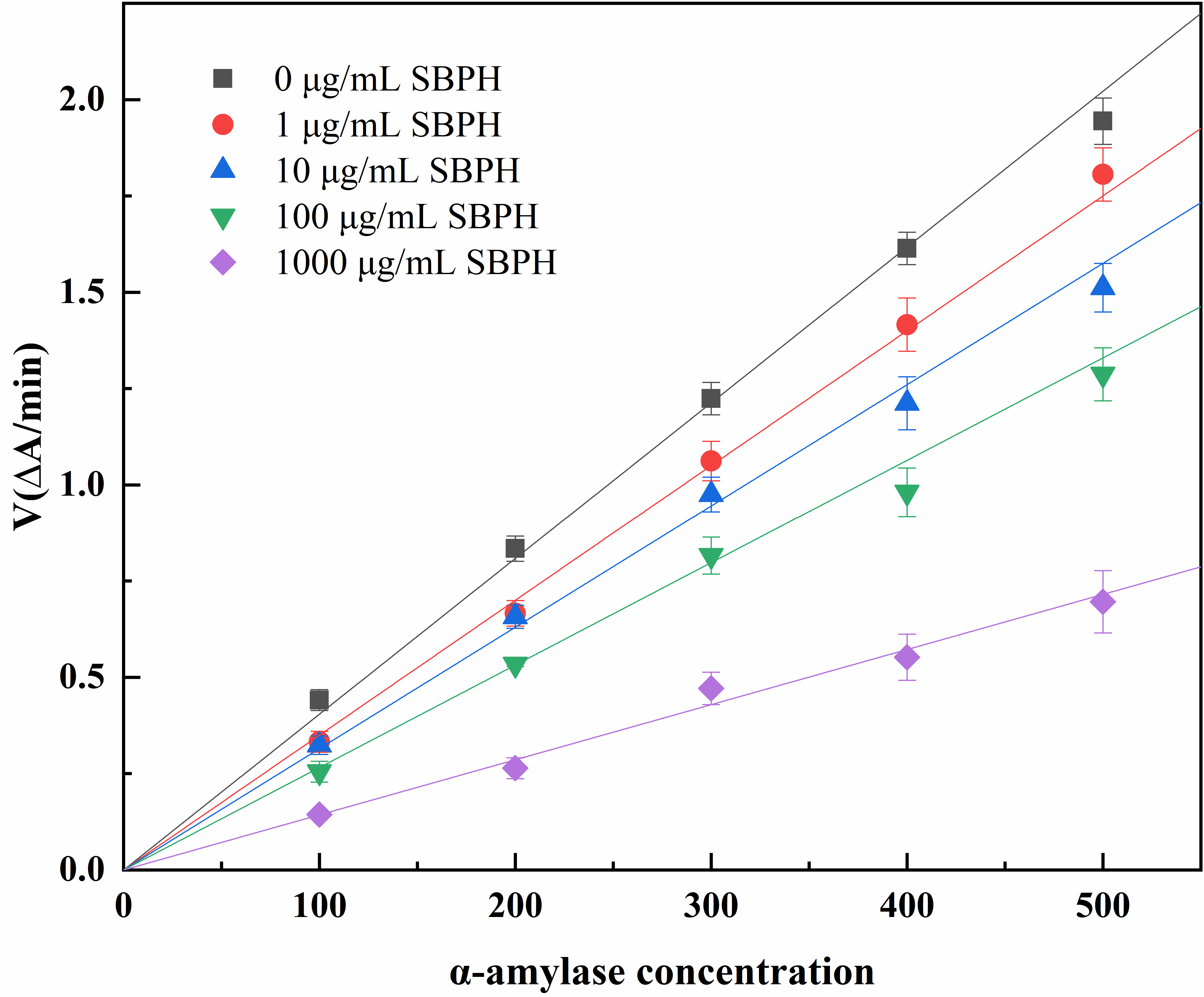

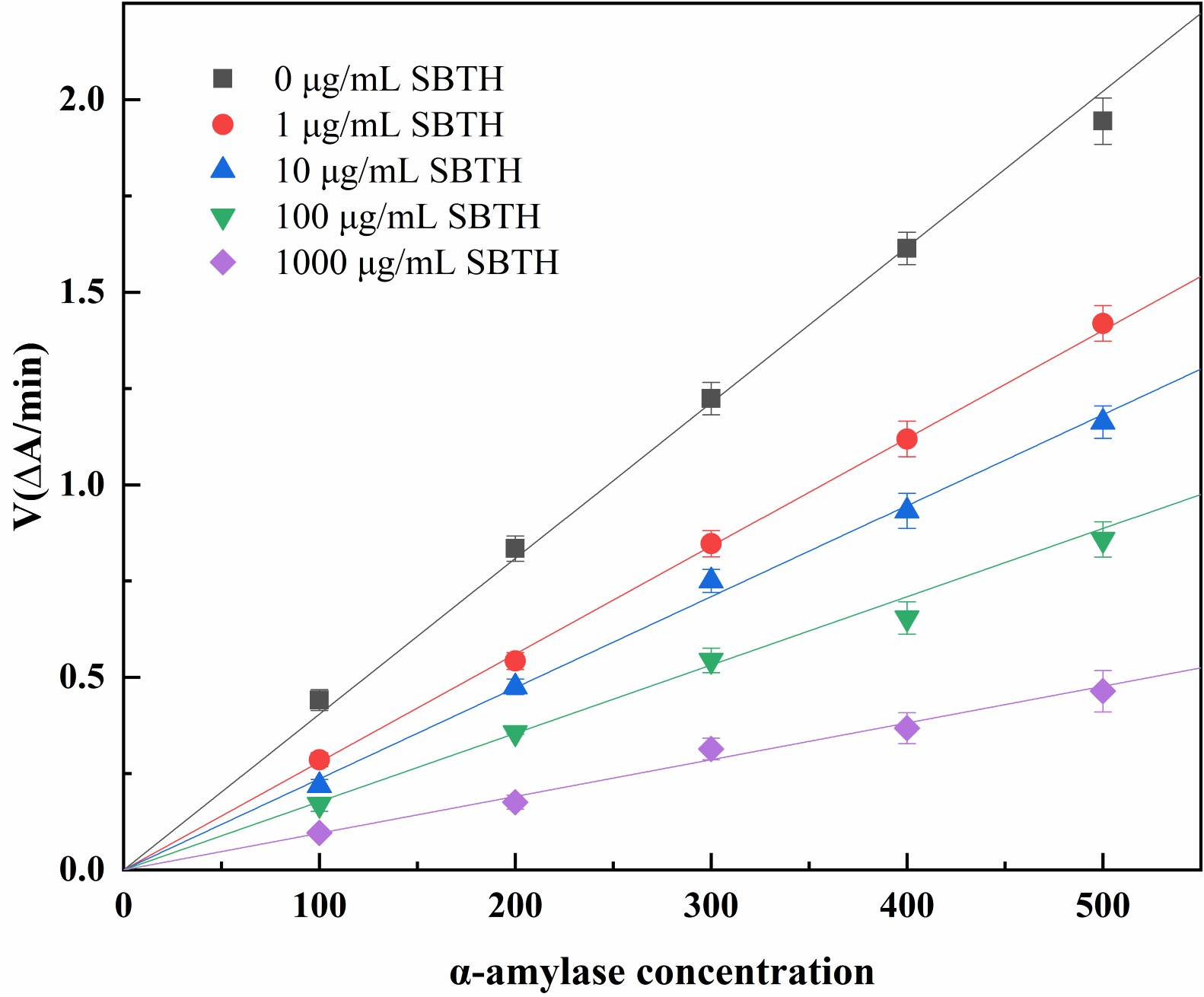

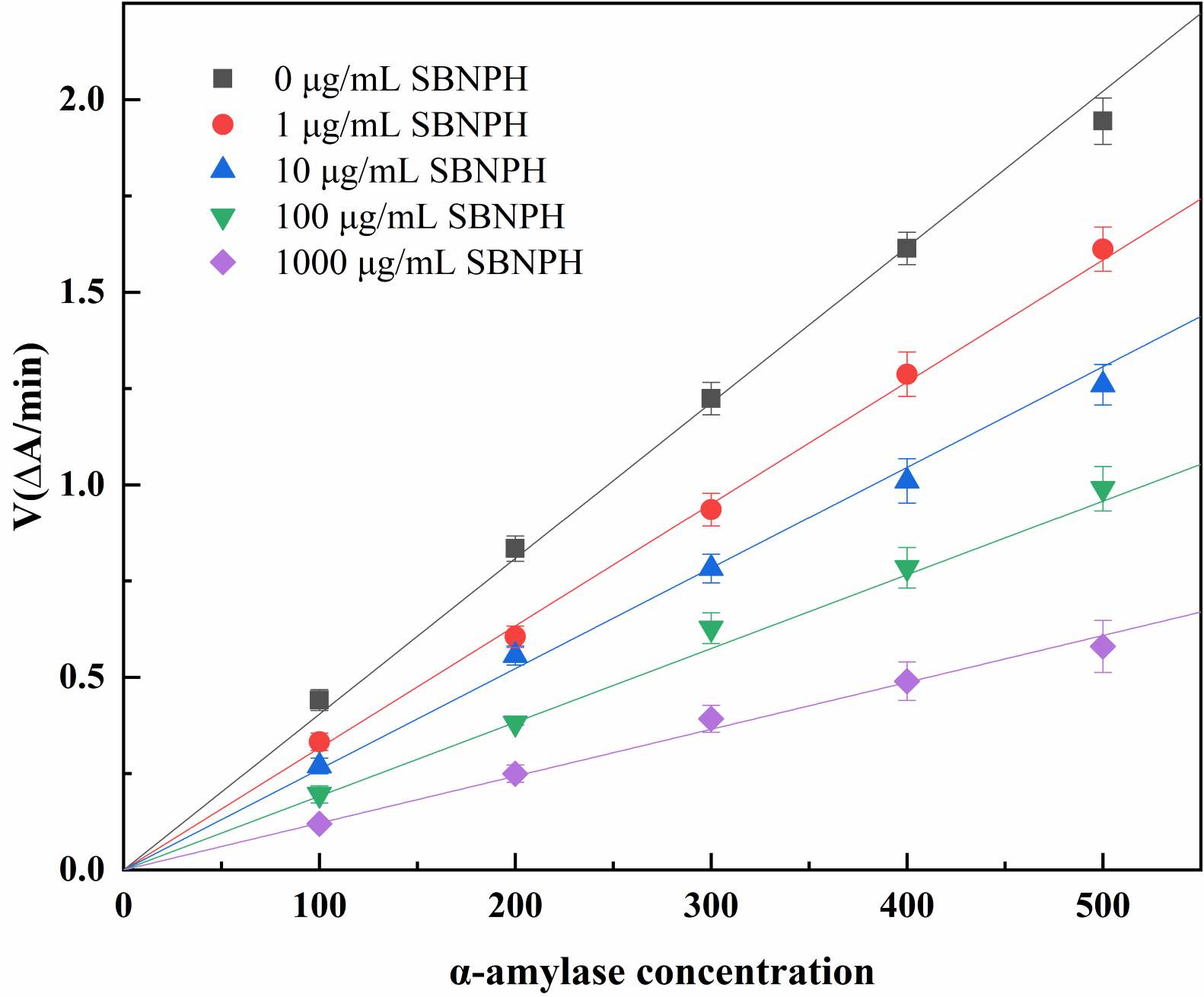

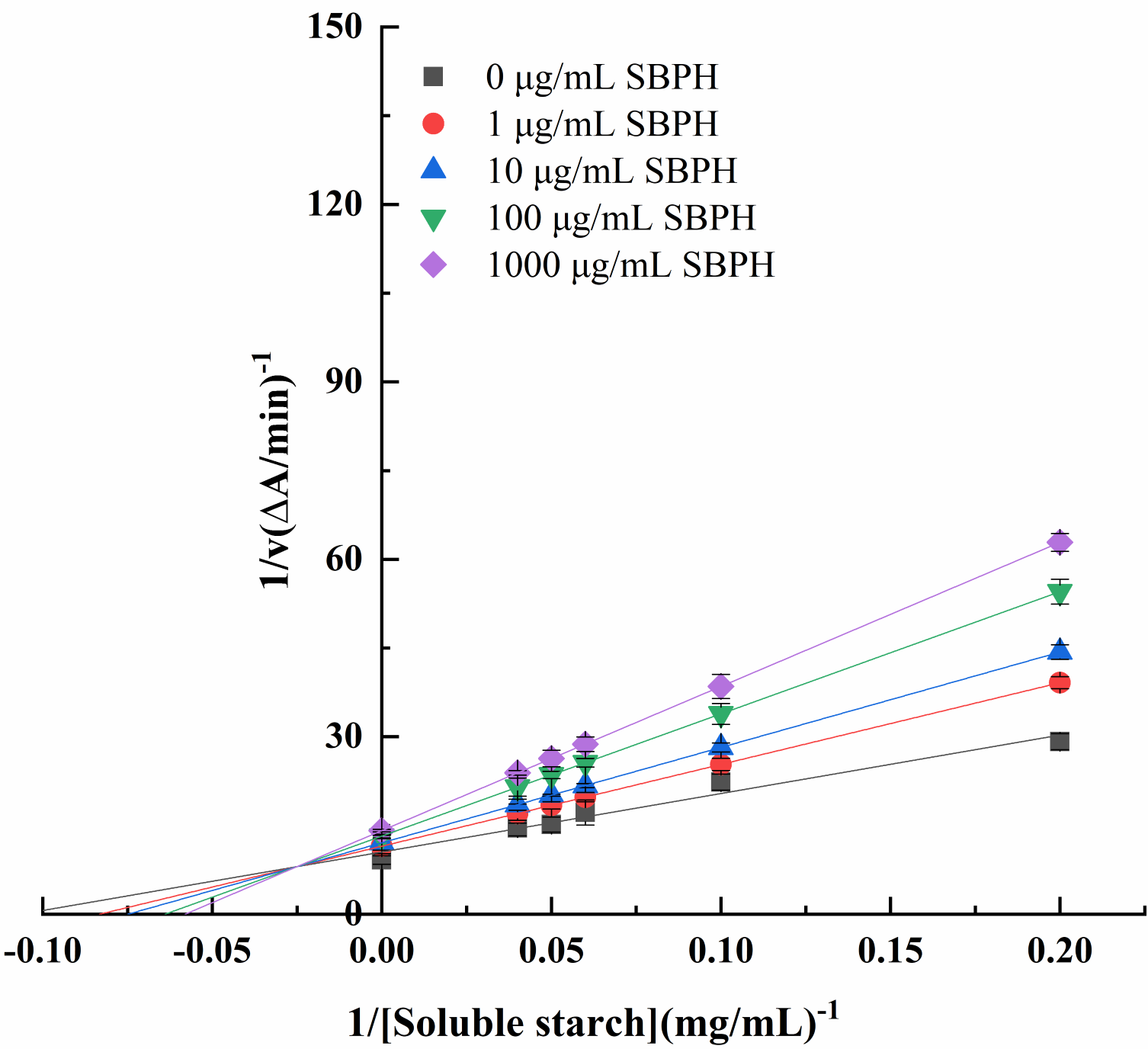

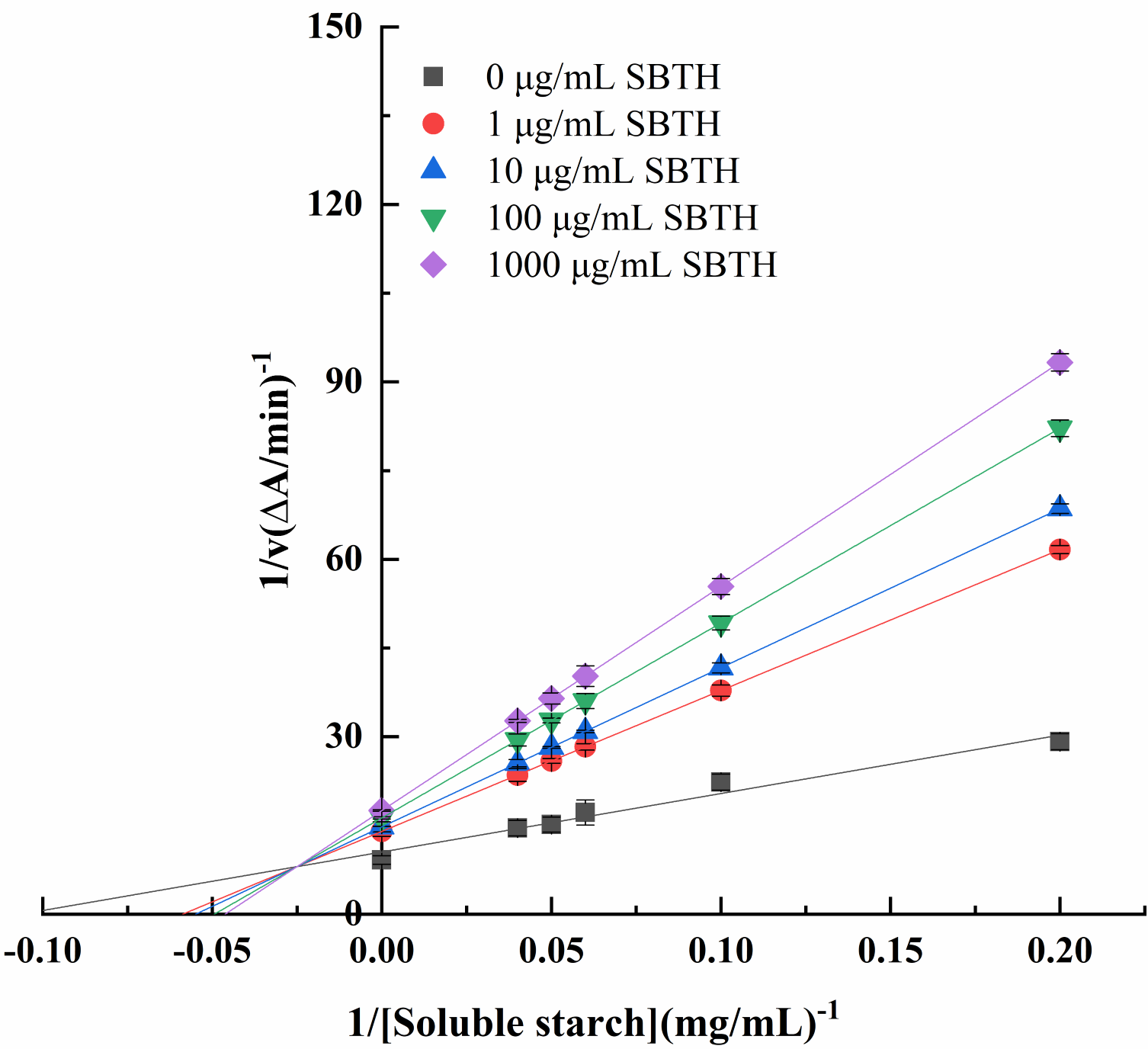

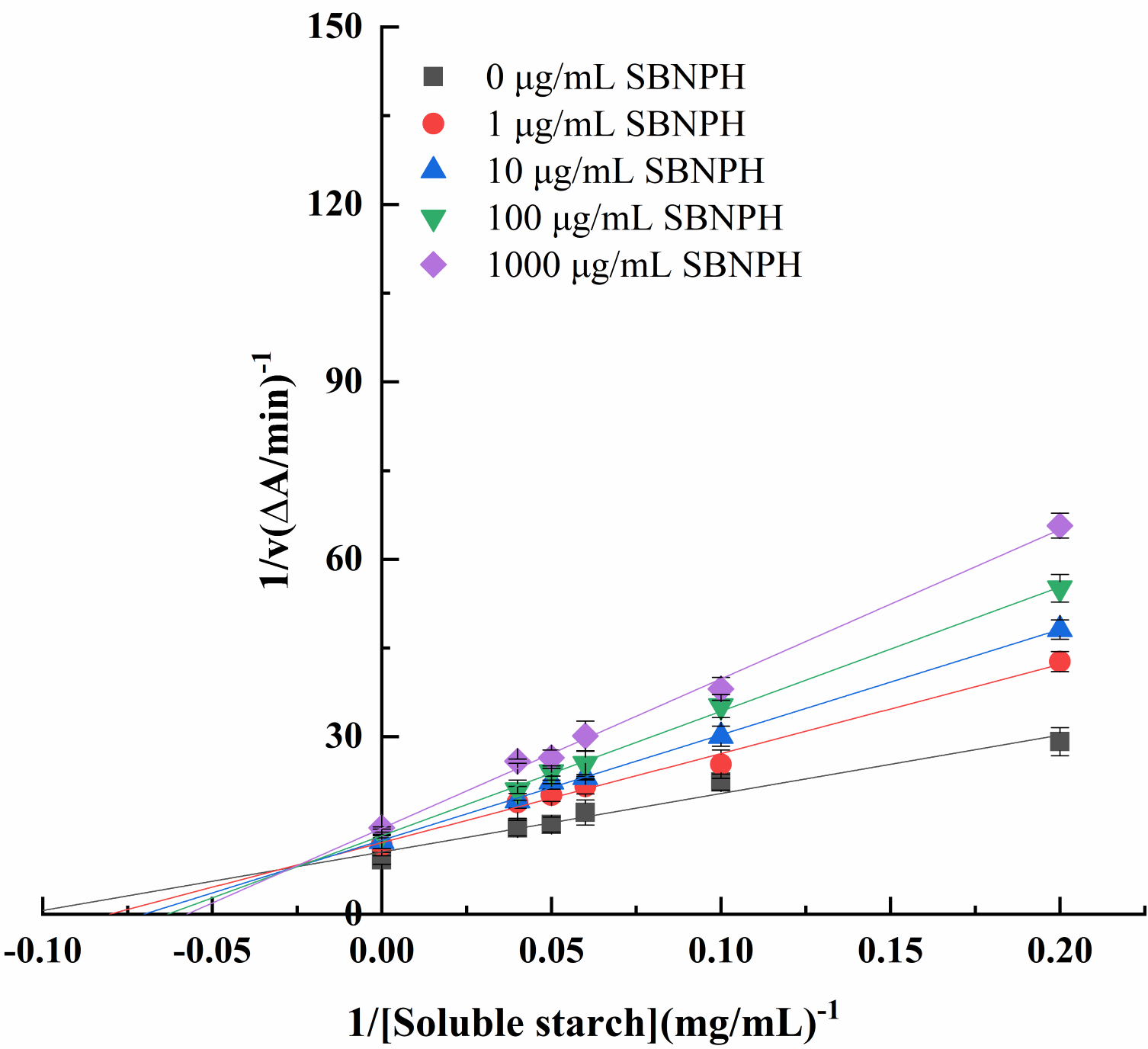


**A_1_**

**A_2_**

**A_3_**

**B_2_**

**B_3_**

**B_1_**


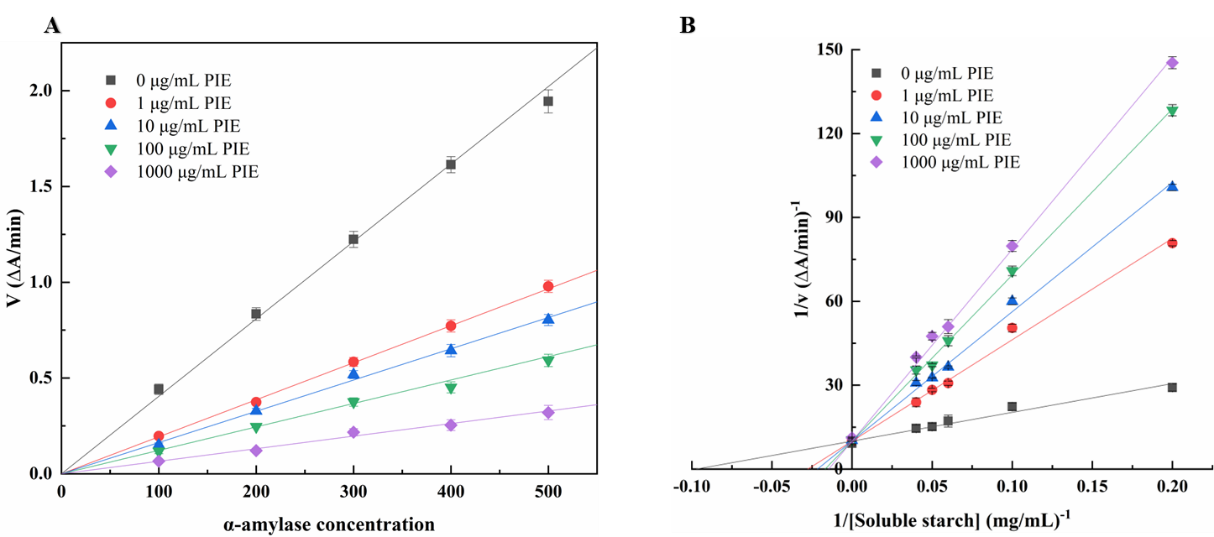
**Figure S3**

**Table S1**

| Sequence | Length | Protein accession | m/z | Intensity |
| --- | --- | --- | --- | --- |
| AAAAVVEFQR | 10 | XP_035606015.1 | 552.29583 | 0.6332 |
| AAAPAPAPEPDVVAAPPALDLSK | 23 | XP_035605134.1 | 723.391438 | 537350000 |
| .  . | .  . | .  . | .  . | .  . |
| IEELEEELEAER | 12 | XP_035613016.1 | 744.856843 | 1717900000 |
| .  . | .  . | .  . | .  . | .  . |
| YYSLGDMTEK | 10 | XP_035632366.1 | 603.770997 | 121540000 |
| YYVTIIDAPGHR | 12 | XP_035651055.1 | 468.91386 | 79033000 |

Note: Detailed information is in a separate Excel sheet.

**Table S2**

| **Enzyme** | **Sample** | **K_I_（mg/mL）** | **K_IS_（mg/mL）** | **Inhibition type** |
| --- | --- | --- | --- | --- |
| **α-amylase** | SBPH | 7.16 | 8.63 | Mixed inhibition |
|  | SBTH | 4.77 | 5.75 | Mixed inhibition |
|  | SBNPH | 5.72 | 6.90 | Mixed inhibition |

Abbreviation: SBSH, salmon bone pepsin hydrolysate; SBTH, salmon bone trypsin hydrolysate; SBNPH, salmon bone neutral protease hydrolysate.

**Table S3**

| **Enzyme** | **Sample** | **K_I_（mg/mL）** | **K_IS_（mg/mL）** | **Inhibition type** |
| --- | --- | --- | --- | --- |
| **α-amylase** | PIE | 3.29 | 3.97 | Competitive  inhibition |

Abbreviation: SBSH, salmon bone pepsin hydrolysate; SBTH, salmon bone trypsin hydrolysate; SBNPH, salmon bone neutral protease hydrolysate.
